# Expression Profile and Role of the *IGF2BP1-3* Genes During Human in vitro Osteogenic Differentiation

**DOI:** 10.1007/s12013-026-02033-z

**Published:** 2026-03-09

**Authors:** Laila Robinson, Hisae Cole, Emma X. Melton, Xinyi Yin, Nica Omandan, Nicole Cubbage, Amy Acosta Cruz, Leah Friedman, Katherine Perez-Nesmith, Morgan Jones, Aldo Omar Pinedo, Sofiya Igorevna Biryukova, Chloe Matz, Lane Bradley, Jillian Lakey, Jaira Ferreira de Vasconcellos

**Affiliations:** Department of Biology, James Madison University, Harrisonburg, VA 22807 USA

**Keywords:** *let-7* microRNAs, IGF2BP1, IGF2BP2, IGF2BP3, bone-marrow mesenchymal stem cells, osteogenic differentiation

## Abstract

MicroRNAs (miRNAs), including the *let-7* family, have been shown to regulate osteogenic differentiation of human mesenchymal stem cells. Moreover, *let-7* miRNAs were upregulated in the muscle tissue of patients who formed ectopic bone compared to patients who did not form ectopic bone after injury. Here we investigated the expression and role of the insulin-like growth factor 2 binding protein genes (*IGF2BP1*, *IGF2BP2*, and *IGF2BP3*), known targets of the *let-7* miRNAs, during human in vitro osteogenic differentiation. *Let-7a* and *let-7b* were slightly upregulated on osteogenic differentiation day 7, downregulated on day 14, and slightly upregulated or unchanged on days 21 and 28. *Let-7d* and *let-7f* were upregulated on days 7, 14, 21, and 28. *IGF2BP1* and *IGF2BP2* were mildly modulated or unchanged on days 7, 21, and 28, and significantly downregulated on day 14, whereas *IGF2BP3* was slightly upregulated on day 7 and slightly downregulated on days 14, 21, and 28. Following *IGF2BP1* knockdown (KD), *ALPL* was upregulated on days 7 and 14, while *RUNX2* and *BGLAP* were mildly upregulated on day 7 and mildly downregulated on day 14. We also observed more calcium deposits on IGF2BP1-KD compared to the control on osteogenic day 14. Finally, downregulation of the *IGF2BP2-3* genes had no major effects on osteogenic differentiation. Altogether, we characterized the differential expression of members from the *let-7*-*IGF2BP* axis during human in vitro osteogenic differentiation and demonstrated a novel mechanistic insight where *IGF2BP1* may enhance the osteogenic commitment of human bone-marrow mesenchymal stem cells.

**Supplementary Information:**

The online version contains supplementary material available at 10.1007/s12013-026-02033-z.

## Introduction

MicroRNAs (miRNAs) are small (containing an average of 22 nucleotides), non-coding RNAs that are highly conserved across evolution and well-known to play a role as post-transcriptional regulators of gene expression, which can happen through a wide variety of mechanisms, including translational repression, messenger RNA (mRNA) cleavage, and deadenylation [1, 2].

The *let-7* family of miRNAs consists of twelve genes encoding nine mature miRNAs in humans (*let-7a*, *let-7b*, *let-7c*, *let-7d*, *let-7e*, *let-7f*, *let-7 g*, *let-7i*, and *miR-98*). *Let-7* miRNAs have been reported as a positive regulator of bone development [3]. Through gain- and loss-of-function experiments, Wei et al. demonstrated that *let-7* promoted osteogenesis and suppressed adipogenesis of human adipose-derived mesenchymal stem cells (hADSCs) through the repression of the high-mobility group AT-hook 2 (*HMGA2*) gene [3]. They also demonstrated that *let-7* promoted ectopic bone formation of hADSCs in vivo [3]. Moreover, *let-7c*-5p was shown to promote the osteogenic differentiation of dental pulp stem cells through the repression of the HMGA2/PI3K/Akt signaling pathway [4]. We have also previously reported a miRNA expression signature for impaired wound-healing and ectopic bone formation that included the upregulation of *let-7d* and *let-7f* miRNAs in humans [5]. Of importance, previous studies identified and validated the *HMGA2* gene as a downstream mRNA target of the *let-7* miRNA family members that play a role in osteogenic differentiation [3, 4].

We hypothesized that additional downstream mRNA targets of the *let-7* miRNAs may play a role in human osteogenic differentiation. Among the multiple potential *let-7* miRNAs targets [6], the insulin-like growth factor 2 mRNA binding protein 1–3 (*IGF2BP1*, *IGF2BP2*, and *IGF2BP3*) are known to have a wide range of cellular functions, including the post-transcriptional regulation of their mRNA target transcripts through effects on RNA stability and translation [7]. Moreover, *let-7* miRNAs have been experimentally validated to directly bind and regulate *IGF2BP1-3* transcripts [6, 8–10]. Of interest, Yan et al. [10]. demonstrated binding and regulation of *let-7b* to *IGF2BP2* during the osteogenic differentiation of human dental pulp stem cells, and the knockdown of *IGF2BP2* promoted osteogenic differentiation as evidenced by increased levels of calcium deposits by Alizarin Red S staining compared to the control. As such, our hypothesis was specifically that *IGF2BP1-3* transcripts would play a role during human osteogenic differentiation.

Here we characterized the expression of selected *let-7* miRNA family members and their downstream mRNA targets, *IGF2BP1*-*3* [6, 8, 9], as well as investigated the potential role of *IGF2BP1*-*3* during human in vitro osteogenic differentiation. The experimental design used in this study was centered around directly targeting the expression of *IGF2BP1*, *IGF2BP2*, or *IGF2BP3* to investigate any effects on osteogenic differentiation in vitro, which allowed us to identify a novel mechanistic insight where *IGF2BP1* may enhance the osteogenic commitment of human bone-marrow mesenchymal stem cells (BM-MSCs).

## Materials and Methods

### Cells and Culture for Cell Maintenance

BM-MSCs were obtained from Genecopoeia (catalog number SL428, lot number G12CL5J1P13, Rockville, MD). Cell culture reagents were purchased from Thermo Fisher Scientific (Carlsbad, CA) unless otherwise stated. BM-MSCs were cultured until confluence in complete growth medium (Dulbecco’s Modified Eagle Medium (DMEM) with 4.5 g/L of D-Glucose supplemented with 10% fetal bovine serum (FBS), 1% Penicillin/Streptomycin, and 1% Amphotericin B. Cells were used for osteogenic differentiation induction experiments between passages 3 and 6. All cell culture procedures were approved by the Institutional Biosafety Committee at James Madison University (protocols # 21-2463 and 24-4579). All experimental protocols were performed following relevant guidelines and regulations.

### Osteogenic Differentiation

BM-MSCs were induced to osteogenic differentiation as previously described [5, 11–13]. Briefly, BM-MSCs were seeded at 5,000 cells/cm^2^ (for 28 days osteogenic differentiation experiments) or 10,000 cells/cm^2^ (for 14 days osteogenic differentiation experiments) and treated for up to 4 weeks with osteogenic medium comprised of DMEM with 4.5 g/L of D-Glucose supplemented with 10% FBS, 1% Penicillin/Streptomycin, 1% Amphotericin B, 10 mM β-glycerol phosphate (Sigma-Aldrich, St. Louis, MO), 50 µg/mL ascorbic acid (Sigma-Aldrich), 10 nM 1α,25-Dihydroxyvitamin D3 (Santa Cruz Biotechnology, Inc., Dallas, TX) and 0.01 µM dexamethasone (Sigma-Aldrich). Osteogenic differentiation started on average 1-day after seeding the cells. In some experiments (as indicated), cells were cultured in complete growth medium consisting of DMEM with 4.5 g/L of D-Glucose supplemented with 10% FBS, 1% Penicillin/Streptomycin, and 1% Amphotericin B for comparison (control).

### Transfection with IGF2BP1, IGF2BP2, or IGF2BP3 siRNAs

The transfection of IGF2BP1, IGF2BP2, or IGF2BP3 siRNAs was performed in BM-MSCs using 25 nM siRNAs (Sigma-Aldrich) and Lipofectamine RNAiMAX (Thermo Fisher Scientific) following the manufacturer’s instructions and compared to a negative (scramble) control siRNA (Thermo Fisher Scientific). RNA samples were collected 48 h after transfection (before osteogenic differentiation) as described below. Additional transfected cells were submitted to osteogenic differentiation for up to 14 days, starting 48 h after transfection (on average, 3 days after seeding the cells). RNA samples were harvested on days 7 and 14 of osteogenic differentiation as described below.

### Alizarin Red S Staining

Following osteogenic induction, BM-MSCs cells were fixed with 0.5% glutaraldehyde (Sigma-Aldrich) and stained with 2% Alizarin Red S at pH 4.2 (Sigma-Aldrich) for evidence of a mineralized matrix, as previously described [5, 12, 13]. Images were captured on the Nikon Eclipse T*i*2 Microscope at the Department of Biology Light Microscopy and Imaging Facility, James Madison University.

### RNA Isolation, Complementary DNA (cDNA) Synthesis, and Quantitative PCR (qRT-PCR) Analysis for Messenger RNA (mRNA) Targets

RNA samples were harvested using Qiazol (Qiagen, Germantown, MD) and purified using the miRNeasy kit (Qiagen) following the manufacturer’s instructions. RNA concentration was measured using a NanoDrop spectrophotometer (Center for Genome and Metagenome Studies, James Madison University). Complementary DNA (cDNA) reverse transcription was performed using the High-Capacity cDNA Reverse Transcription Kit (Thermo Fisher Scientific) following the manufacturer’s instructions. Quantitative PCR (qRT-PCR) analysis was performed in a Bio-Rad CFX384 real-time PCR system (Bio-Rad, Hercules, CA) using TaqMan™ Universal PCR Master Mix (Thermo Fisher Scientific) or SsoAdvanced Universal SYBR Green Supermix (Bio-Rad) following the manufacturer’s instructions. The following commercially available Assay-on-Demand gene expression products (Thermo Fisher Scientific) were used: *IGF2BP1* (Hs00977566_m1), *IGF2BP2* (Hs00538956_m1), and *IGF2BP3* (Hs01122560_g1). Gene expression was normalized using *GAPDH* (Hs02758991_g1). Cycling conditions when using the Assay-on-Demand gene expression products were as follows: 50°C for 2 minutes, 95°C for 10 minutes, and 40 cycles of 95°C for 15 seconds followed by 60°C for 1 minute. Additionally, the osteogenic (*ALPL*, *RUNX2*, and *BGLAP*) and *GAPDH* primers used in this study have been previously described [13] or were newly designed as follows: *RUNX2*-Forward: 5’-agaaggcacagacagaagct-3’, *RUNX2*-Reverse: 5’-tgcctggggtctgtaatctg-3’, *BGLAP*-Forward: 5’-gcagcgaggtagtgaagaga-3’, *BGLAP*-Reverse: 5’-ctggagaggagcagaactgg-3’. Cycling conditions when using SYBR Green Supermix (Bio-Rad) were as follows: 95 °C for 30 s, and 40 cycles of 95 °C for 15 s followed by 60 °C for 30 s. Melting curve analysis was performed from 65 °C to 95 °C with 0.5 °C increments and 5 s/step. Relative gene expression was calculated using the 2^−∆∆Ct^ method [14]. Reactions were performed in triplicate.

### RNA Isolation, cDNA Synthesis, and qRT-PCR Analysis for miRNA Targets

RNA samples were harvested using Qiazol (Qiagen) and purified using the miRNeasy kit (Qiagen) following the manufacturer’s instructions. RNA concentration was measured using a NanoDrop spectrophotometer (Center for Genome and Metagenome Studies, James Madison University). Complementary DNA (cDNA) reverse transcription was performed using the TaqMan™ MicroRNA Reverse Transcription Kit (Thermo Fisher Scientific) following the manufacturer’s instructions. Quantitative PCR (qRT-PCR) analysis was performed in a Bio-Rad CFX384 real-time PCR system (Bio-Rad) using Taqman™ MicroRNA assay (Thermo Fisher Scientific) for the following miRNA targets: hsa-*let-7a* (assay ID:000377), hsa-*let-7b* (assay ID:002619), hsa-*let-7d* (assay ID:002283), and hsa-*let-7f* (assay ID:000382). Gene expression was normalized using *miR-16* (assay ID:000391). Cycling conditions when using the Taqman™ MicroRNA assay products were as follows: 50 °C for 2 min, 95 °C for 10 min, and 40 cycles of 95 °C for 15 s followed by 60 °C for 1 min. Relative gene expression was calculated using the 2^−∆∆Ct^ method [14]. Reactions were performed in triplicate.

### Western Blot

Protein samples were harvested using RIPA buffer containing protease inhibitors (both from ThermoFisher Scientific). Protein analyses were performed following standard protocols and as previously described [15]. Briefly, 10–15 µg of total protein extracts were separated by gel electrophoresis using a NuPAGE^®^ 4–12% Bis-Tris Gel (Thermo Fisher Scientific), followed by transfer onto a nitrocellulose membrane (Bio-Rad). Membranes were incubated with antibodies against IGF2BP1 (Cell Signaling Technology), IGF2BP2 (Cell Signaling Technology), or IGF2BP3 (Abcam) as indicated. Beta-actin (Cell Signaling Technology) was used as a loading control. Detection was performed by incubation with a horseradish peroxidase-conjugated rabbit secondary antibody (EMD/Millipore, Burlington, MA; 1:10,000) followed by Immobilon Western Chemiluminescent HRP Substrate Kit (Millipore). Western blot signal acquisition was performed with a ChemiDoc™ Imaging System (Bio-Rad).

### Microarray Gene Expression Analyses

Publicly available gene expression datasets (series GSE48129 and GSE94683) from patients with different types of ectopic bone formation were selected from the Gene Expression Omnibus data repository (https://www.ncbi.nlm.nih.gov/geo/). Datasets were analyzed using the NCBI interactive web tool GEO2R to generate a list of differentially expressed genes [16]. The false discovery rate (FDR)-adjusted p-value was ⩽ 0.05.

### Statistical Analysis

Replicates were expressed as mean *±* standard deviation values from at least three independent experiments. Significance was calculated by a two-tailed Student’s t-test. Data visualization was performed using GraphPad Prism 10 (GraphPad Software, Boston, MA).

## Results

### *IGF2BP1-3* Genes are Downregulated in Publicly Available Gene Expression Datasets from Patients with Ectopic Bone Formation

The *let-7* miRNAs have been reported to promote in vitro osteogenic differentiation in human stromal/mesenchymal stem cells through the repression of the *HMGA2* gene [3]. Moreover, we previously reported a human miRNA expression signature for ectopic bone formation that included the upregulation of *let-7d* and *let-7f* miRNAs [5]. Based on these findings, we hypothesized that additional downstream mRNA targets of the *let-7* miRNAs may play a role in human osteogenic differentiation. To investigate this, we searched for the expression levels of experimentally validated *let-7* miRNAs’ targets [6, 8–10] in publicly available gene expression datasets at the Gene Expression Omnibus data repository (https://www.ncbi.nlm.nih.gov/geo/). We observed consistent low expression levels (ranging from 0.135 to -3.165) of the *IGF2BP1-3* genes in two independent publicly available gene expression studies from patients with different types of ectopic bone formation (Supplementary Material 1).

### The *let-7-IGF2BP1-3* Axis is Modulated During Human in vitro Osteogenic Differentiation

**Fig. 1:**
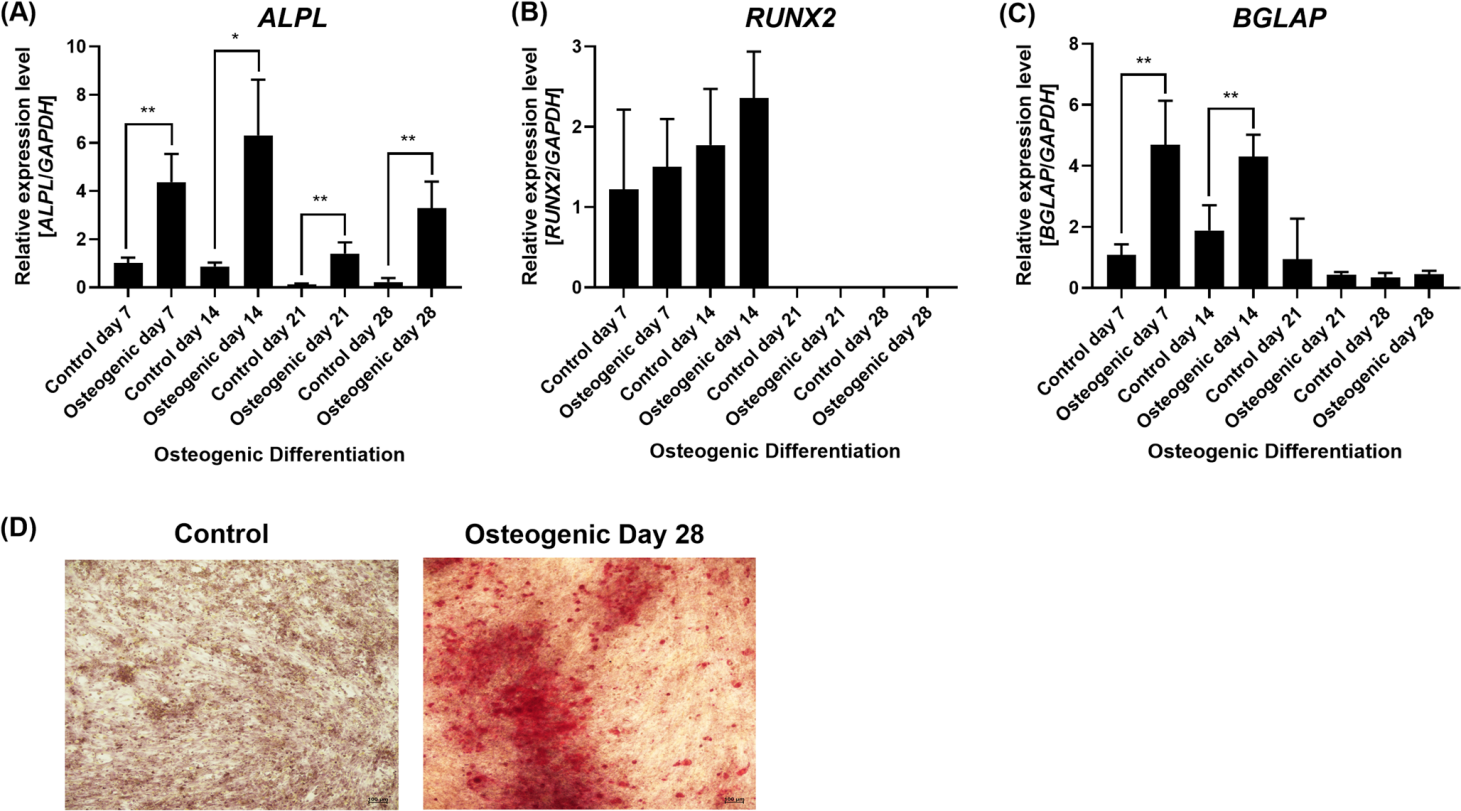
Characterization of the osteogenic differentiation potential from the bone-marrow mesenchymal stem cells (BM-MSCs) used in this study. Relative expression levels of the osteogenic genes **(A)** *ALPL*, **(B)** *RUNX2*, and **(C)** *BGLAP* were investigated by qRT-PCR during osteogenic differentiation on days 7, 14, 21, and 28. Gene expression was normalized using *GAPDH*. Expression levels were calculated relative to the control on day 7. Mean value *±* standard deviation of at least three independent experiments. **(D)** Osteogenic differentiation was confirmed by Alizarin Red S staining on day 28. Representative images are shown from control BM-MSCs (cultured in growth media) compared to BM-MSCs induced to osteogenic differentiation (cultured in osteogenic media). BM-MSCs cultured in osteogenic media demonstrated increased calcium deposits, which is evidence of the formation of a mineralized matrix that stained positively for Alizarin Red S. Images are at 10X magnification. *p ≦ 0.05; **p ≦ 0.01; two-tailed Student’s t-test compared to each respective control

These results led us to hypothesize that the *IGF2BP1-3* genes, which are experimentally validated downstream mRNA targets of the *let-7* miRNAs [6, 8–10], may play a role in human osteogenic differentiation. To investigate that we induced BM-MSCs into osteogenic differentiation for 28 days as previously described [5, 11–13]. All the qRT-PCR relative gene expression results were compared to the growth media control at day 7 of culture, and all the statistical significance was compared to each respective timepoint control. Osteogenic differentiation was confirmed by the upregulation of *ALPL* (Control day 14: 0.9 *±* 0.2; Osteogenic day 14: 6.3 *±* 2.3; *p* = 0.02), *RUNX2* (Control day 14: 1.8 *±* 0.7; Osteogenic day 14: 2.4 *±* 0.6; *p* = 0.41) and *BGLAP* (Control day 14: 1.9 *±* 0.8; Osteogenic day 14: 4.3 *±* 0.7; *p* = 0.01) genes at osteogenic differentiation day 14 (Fig. 1A-C), which are known hallmarks of osteogenic differentiation. In addition, Alizarin Red S staining on day 28 demonstrated evidence of a mineralized matrix in BM-MSCs treated with osteogenic media compared to control cells treated with growth media (Fig. 1D).

**Fig. 2:**
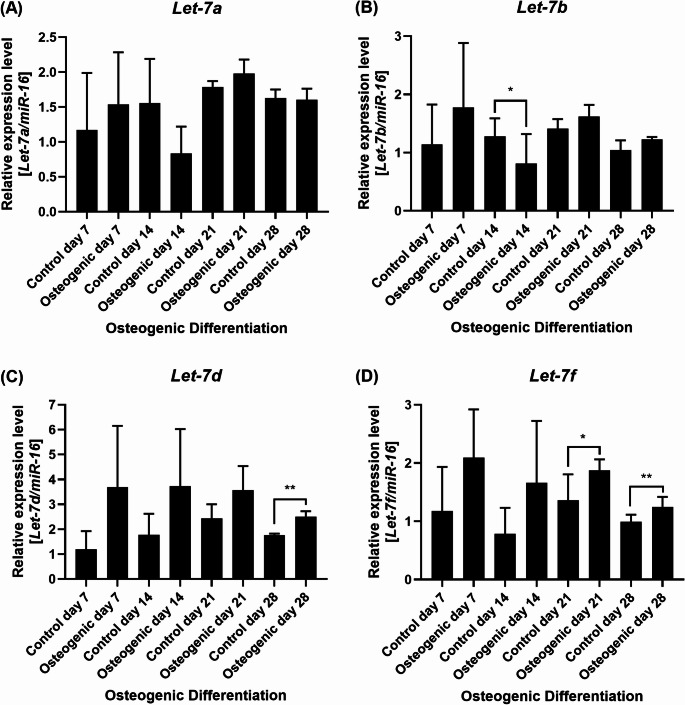
*Let*-*7* miRNAs are modulated during human in vitro osteogenic differentiation. Relative expression levels of **(A)** *let-7a*, **(B)** *let-7b*, **(C)** *let-7d*, and **(D)** *let-7f* were investigated by qRT-PCR during osteogenic differentiation days 7, 14, 21, and 28. Gene expression was normalized using *miR-16*. Expression levels were calculated relative to the control on day 7. Mean value *±* standard deviation of at least three independent experiments. *p ≦ 0.05; **p ≦ 0.01; two-tailed Student’s t-test compared to each respective control

Then, we characterized the expression profile of selected *let-7* miRNAs during human in vitro osteogenic differentiation. Selection of the *let-7* miRNAs investigated in this study was based on the following parameters: (i) *let-7a* is the *let-7* miRNA family member most conserved throughout evolution [17, 18], (ii) *let-7b* was shown to be upregulated during estradiol-17*β*-induced osteogenic differentiation [19] and downregulated during the osteogenic differentiation of stem cells from apical papilla [20], (iii) *let-7d* as well as *let-7f* were previously reported to be upregulated in patients who developed ectopic bone compared with patients who did not develop ectopic bone after traumatic extremity injury [5], and (iv) *let-7a*, *let-7b* and *let-7f* (among other miRNAs, including members of the *let-7* family) were shown to be upregulated following osteogenic differentiation of human unrestricted somatic stem cells compared to undifferentiated controls [21]. In this study, *let-7a* was slightly upregulated on osteogenic differentiation day 7 (Control day 7: 1.2 *±* 0.8; Osteogenic day 7: 1.5 *±* 0.7; *p* = 0.53), slightly downregulated on day 14 (Control day 14: 1.6 *±* 0.6; Osteogenic day 14: 0.8 *±* 0.4; *p* = 0.09), slightly upregulated on day 21 (Control day 21: 1.8 *±* 0.1; Osteogenic day 21: 2.0 *±* 0.2; *p* = 0.06), and unchanged on day 28 (Control day 28: 1.6 *±* 0.1; Osteogenic day 28: 1.6 *±* 0.2; *p* = 0.86, respectively, Fig. 2A). *Let-7b* was slightly upregulated on day 7 (Control day 7: 1.1 *±* 0.7; Osteogenic day 7: 1.8 *±* 1.1; *p* = 0.12), downregulated on day 14 (Control day 14: 1.3 *±* 0.3; Osteogenic day 14: 0.8 *±* 0.5; *p* = 0.03) and slightly upregulated on days 21 and 28 (Control day 21: 1.4 *±* 0.2; Osteogenic day 21: 1.6 *±* 0.2; *p* = 0.18 and Control day 28: 1.0 *±* 0.2; Osteogenic day 28: 1.2 *±* 0.0; *p* = 0.15, respectively, Fig. 2B). *Let-7d* was mildly upregulated on day 7 (Control day 7: 1.2 *±* 0.7; Osteogenic day 7: 3.7 *±* 2.5; *p* = 0.11), day 14 (Control day 14: 1.8 *±* 0.8; Osteogenic day 14: 3.7 *±* 2.3; *p* = 0.23), day 21 (Control day 21: 2.4 *±* 0.6; Osteogenic day 21: 3.6 *±* 1.0; *p* = 0.06) and day 28 (Control day 28: 1.8 *±* 0.1; Osteogenic day 28: 2.5 *±* 0.2; *p* = 0.004; respectively, Fig. 2C). Similarly, *let-7f* was mildly upregulated on day 7 (Control day 7: 1.2 *±* 0.8; Osteogenic day 7: 2.1 *±* 0.8; *p* = 0.13), day 14 (Control day 14: 0.8 *±* 0.4; Osteogenic day 14: 1.7 *±* 1.1; *p* = 0.41), day 21 (Control day 21: 1.4 *±* 0.4; Osteogenic day 21: 1.9 *±* 0.2; *p* = 0.04) and day 28 (Control day 28: 1.0 *±* 0.1; Osteogenic day 28: 1.2 *±* 0.2; *p* = 0.01; respectively, Fig. 2D).

**Fig. 3:**
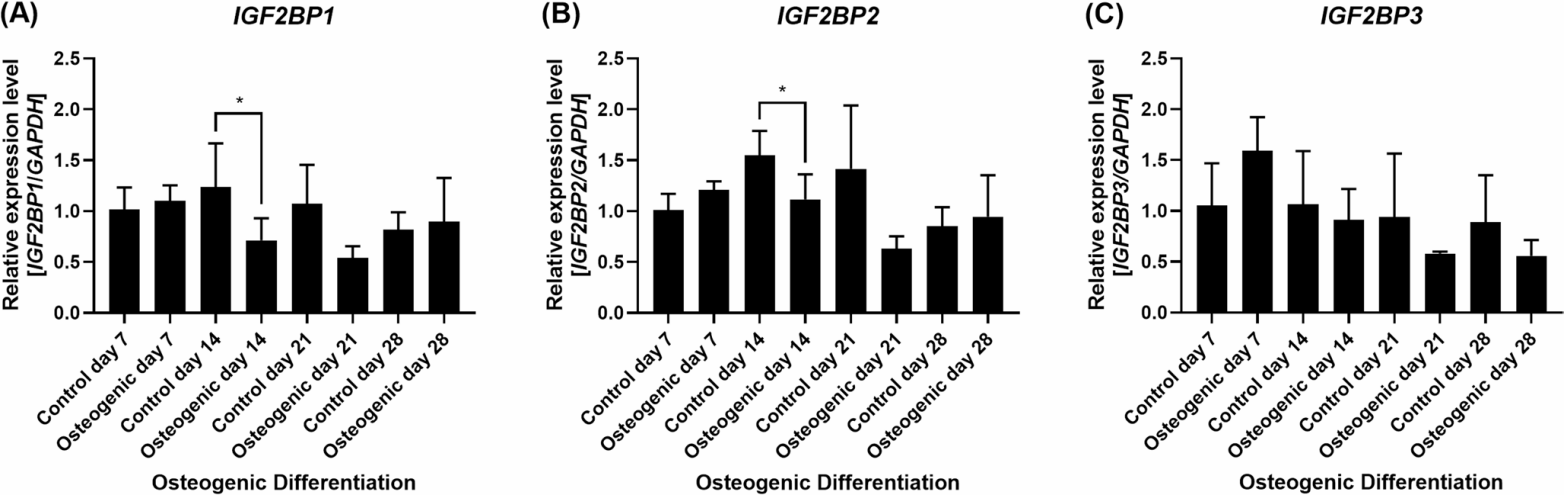
The *IGF2BP1-3* genes are modulated during human in vitro osteogenic differentiation. Relative expression levels of **(A)** *IGF2BP1*, **(B)** *IGF2BP2*, and **(C)** *IGF2BP3* were investigated by qRT-PCR during osteogenic differentiation on days 7, 14, 21, and 28. Gene expression was normalized using *GAPDH*. Expression levels were calculated relative to the control on day 7. Mean value *±* standard deviation of at least three independent experiments. *p ≦ 0.05; two-tailed Student’s t-test compared to each respective control

Finally, we characterized the expression profile of the *IGF2BP1-3* genes during human in vitro osteogenic differentiation. *IGF2BP1* and *IGF2BP2* were mildly modulated or unchanged on osteogenic differentiation days 7, 21 and 28 (*IGF2BP1*: Control day 7: 1.0 *±* 0.2; Osteogenic day 7: 1.1 *±* 0.2; *p* = 0.53; Control day 21: 1.1 *±* 0.4; Osteogenic day 21: 0.5 *±* 0.1; *p* = 0.10; Control day 28: 0.8 *±* 0.2; Osteogenic day 28: 0.9 *±* 0.4; *p* = 0.72 and *IGF2BP2*: Control day 7: 1.0 *±* 0.2; Osteogenic day 7: 1.2 *±* 0.1; *p* = 0.08; Control day 21: 1.4 *±* 0.6; Osteogenic day 21: 0.6 *±* 0.1; *p* = 0.10; Control day 28: 0.9 *±* 0.2; Osteogenic day 28: 0.9 *±* 0.4; *p* = 0.50), and significantly downregulated on day 14 (*IGF2BP1*: Control day 14: 1.2 *±* 0.4; Osteogenic day 14: 0.7 *±* 0.2; *p* = 0.02 and *IGF2BP2*: Control day 14: 1.5 *±* 0.2; Osteogenic day 14: 1.1 *±* 0.2; *p* = 0.02; Fig. 3A-B). Interestingly, *IGF2BP3* was mildly upregulated on day 7 (Control day 7: 1.1 *±* 0.4; Osteogenic day 7: 1.6 *±* 0.3; *p* = 0.09), and slightly downregulated on days 14, 21 and 28 (Control day 14: 1.1 *±* 0.5; Osteogenic day 14: 0.9 *±* 0.3; *p* = 0.34; Control day 21: 0.9 *±* 0.6; Osteogenic day 21: 0.6 *±* 0.0; *p* = 0.41; Control day 28: 0.9 *±* 0.5; Osteogenic day 28: 0.6 *±* 0.2; *p* = 0.31; Fig. 3C). Altogether, these results demonstrate a unique expression pattern for different *let-7* miRNA family members, as well as that the *IGF2BP1-3* genes are slightly differentially modulated during human in vitro osteogenic differentiation.

### Downregulation of *IGF2BP1* may Enhance the Osteogenic Commitment of Human BM-MSCs Induced to in vitro Osteogenic Differentiation

**Fig. 4:**
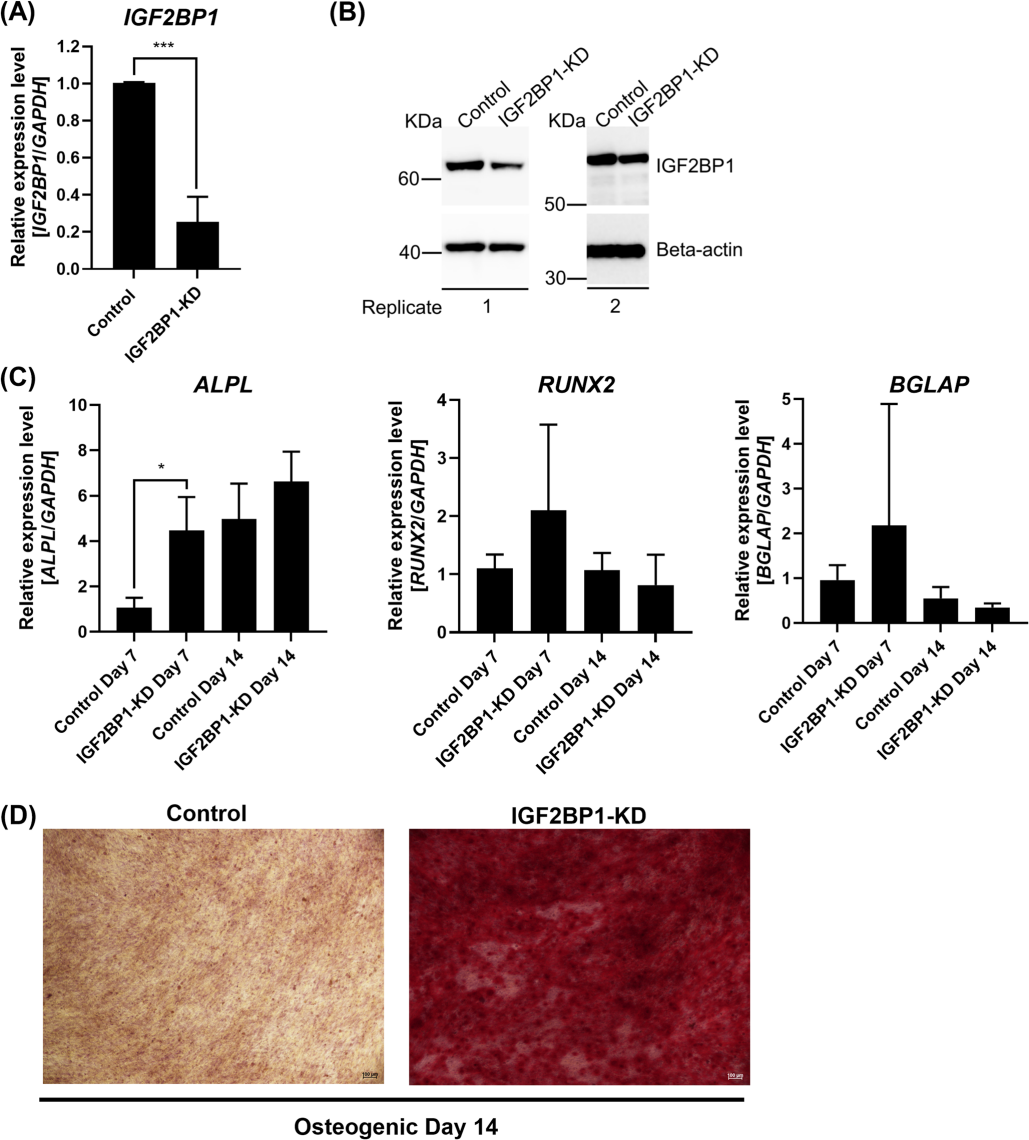
Knockdown of *IGF2BP1* may enhance the osteogenic commitment of human BM-MSCs induced to in vitro osteogenic differentiation. The knockdown of *IGF2BP1* was confirmed by **(A)** qRT-PCR and **(B)** Western blot analyses. Gene expression was normalized using *GAPDH*. Mean value *±* standard deviation of at least three independent experiments. Representative images are shown from transfection control (Control) and *IGF2BP1* knockdown (IGF2BP1-KD) from two independent experiments. Beta-actin was used as a loading control. Molecular weight is shown in Kilodaltons (KDa). **(C)** Relative expression levels of the osteogenic genes *ALPL*, *RUNX2*, and *BGLAP* were investigated by qRT-PCR during osteogenic differentiation on days 7 and 14. Gene expression was normalized using *GAPDH*. Expression levels were calculated relative to the control on day 7. Mean value *±* standard deviation of at least three independent experiments. **(D)** Changes in osteogenic differentiation were investigated by Alizarin Red S staining on day 14. Representative images are shown from transfection control (Control) and *IGF2BP1* knockdown (IGF2BP1-KD) cultured with osteogenic media. Images are at 10X magnification. *p ≦ 0.05; ***p ≦ 0.001; two-tailed Student’s t-test compared to each respective control

To investigate if the *IGF2BP1* gene plays a role during the early stages of human in vitro osteogenic differentiation, we performed the knockdown of the *IGF2BP1* gene (IGF2BP1-KD) in BM-MSCs, followed by 14 days of osteogenic differentiation. The knockdown of *IGF2BP1* was confirmed by qRT-PCR and Western blot compared to a scramble control 48 h after transfection (qRT-PCR: Control: 1.0 *±* 0.0; IGF2BP1-KD: 0.3 *±* 0.1; *p* = 0.001; Fig. 4A-B). IGF2BP1-KD was observed to upregulate the expression of the *ALPL* gene on days 7 and 14 of osteogenic differentiation (Day 7: Control: 1.1 *±* 0.4; IGF2BP1-KD: 4.5 *±* 1.5; *p* = 0.02 and Day 14: Control: 5.0 *±* 1.6; IGF2BP1-KD: 6.6 *±* 1.3; *p* = 0.29; Fig. 4C), while *RUNX2* and *BGLAP* were slightly upregulated on day 7 and slightly downregulated on day 14 compared to the control (*RUNX2*: Day 7: Control: 1.1 *±* 0.2; IGF2BP1-KD: 2.1 *±* 1.5; *p* = 0.37 and Day 14: Control: 1.1 *±* 0.3; IGF2BP1-KD: 0.8 *±* 0.5; *p* = 0.50, and *BGLAP*: Day 7: Control: 1.0 *±* 0.3; IGF2BP1-KD: 2.2 *±* 2.7; *p* = 0.51 and Day 14: Control: 0.6 *±* 0.3; IGF2BP1-KD: 0.3 *±* 0.1; *p* = 0.07; Fig. 4C). Finally, Alizarin Red S staining was performed to identify calcium deposits on osteogenic differentiation day 14. Interestingly, more calcium deposits were observed in the IGF2BP1-KD compared to the control, which may suggest an enhanced osteogenic commitment upon IGF2BP1-KD on day 14 of osteogenic differentiation **(**Fig. 4D).

### Downregulation of *IGF2BP2-3* Genes has no Major Effects on Human in vitro Osteogenic Differentiation

**Fig. 5:**
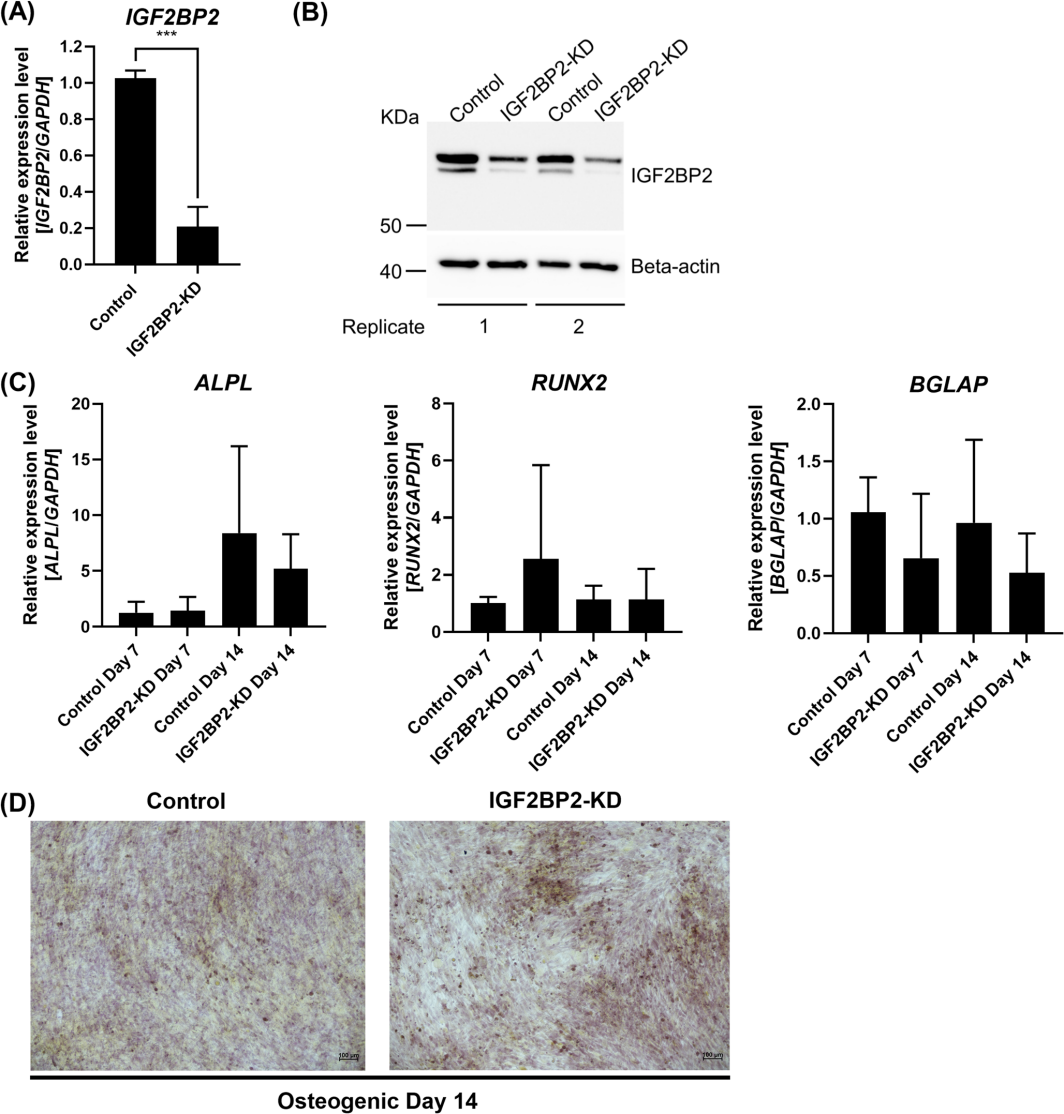
Knockdown of *IGF2BP2* mildly downregulates the expression of *ALPL* in human BM-MSCs induced to in vitro osteogenic differentiation on day 14. The knockdown of *IGF2BP2* was confirmed by **(A)** qRT-PCR and **(B)** Western blot analyses. Gene expression was normalized using *GAPDH*. Mean value *±* standard deviation of at least three independent experiments. Representative images are shown from transfection control (Control) and *IGF2BP2* knockdown (IGF2BP2-KD) from two independent experiments. Beta-actin was used as a loading control. Molecular weight is shown in Kilodaltons (KDa). **(C)** Relative expression levels of the osteogenic genes *ALPL*, *RUNX2*, and *BGLAP* were investigated by qRT-PCR during osteogenic differentiation on days 7 and 14. Gene expression was normalized using *GAPDH*. Expression levels were calculated relative to the control on day 7. Mean value *±* standard deviation of at least three independent experiments. **(D)** Changes in osteogenic differentiation were investigated by Alizarin Red S staining on day 14. Representative images are shown from transfection control (Control) and *IGF2BP2* knockdown (IGF2BP2-KD) cultured with osteogenic media. Images are at 10X magnification. ***p ≦ 0.001; two-tailed Student’s t-test compared to each respective control

Similarly, to investigate if the *IGF2BP2* and *IGF2BP3* genes play a role during the early stages of human in vitro osteogenic differentiation, we performed the knockdown of the *IGF2BP2* (IGF2BP2-KD) and the *IGF2BP3* (IGF2BP3-KD) genes in BM-MSCs, followed by 14 days of osteogenic differentiation. The knockdown of *IGF2BP2* was confirmed by qRT-PCR and Western blot compared to the scramble control 48 h after transfection (qRT-PCR: Control: 1.0 *±* 0.0; IGF2BP2-KD: 0.2 *±* 0.1; *p* = 0.001; Fig. 5A-B). IGF2BP2-KD was observed to slightly downregulate the expression of the *ALPL* gene on day 14 of osteogenic differentiation (Day 7: Control: 1.3 *±* 1.0; IGF2BP2-KD: 1.4 *±* 1.2; *p* = 0.90 and Day 14: Control: 8.4 *±* 7.8; IGF2BP2-KD: 5.2 *±* 3.1; *p* = 0.49; Fig. 5C), mildly upregulate *RUNX2* on day 7 (Day 7: Control: 1.0 *±* 0.2; IGF2BP2-KD: 2.5 *±* 3.3; *p* = 0.27) while day 14 remained unchanged (Day 14: Control: 1.1 *±* 0.5; IGF2BP2-KD: 1.1 *±* 1.1; *p* = 0.99; Fig. 5C), and mildly downregulate *BGLAP* on days 7 and 14 compared to the scramble control (Day 7: Control: 1.1 *±* 0.3; IGF2BP2-KD: 0.7 *±* 0.6; *p* = 0.23 and Day 14: Control: 1.0 *±* 0.7; IGF2BP2-KD: 0.5 *±* 0.3; *p* = 0.11; Fig. 5C). Finally, no differences were observed in the Alizarin Red S staining from IGF2BP2-KD compared to the control on osteogenic differentiation day 14 (Fig. 5D).

**Fig. 6:**
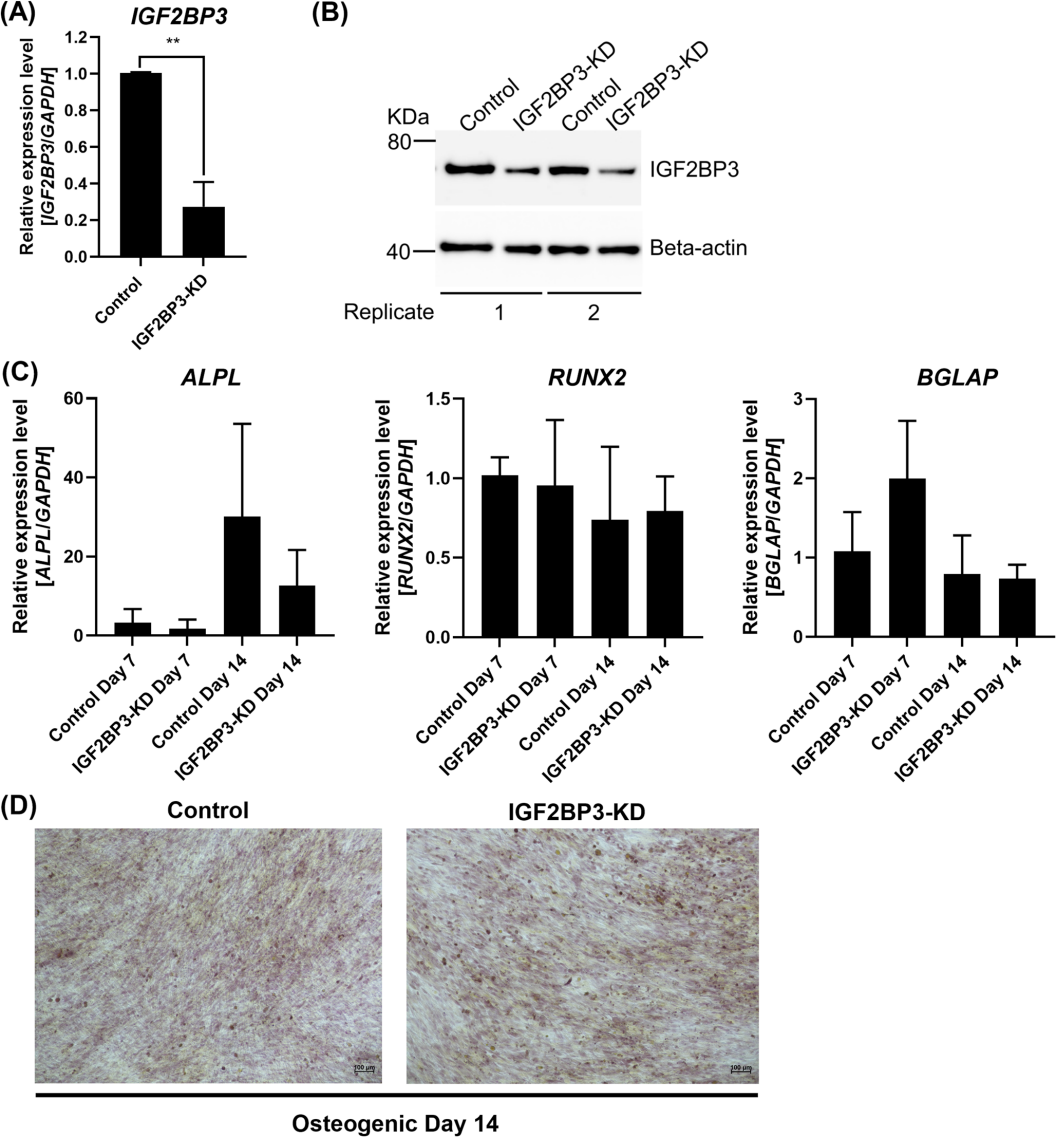
Knockdown of *IGF2BP3* mildly downregulates the expression of *ALPL* in human BM-MSCs induced to in vitro osteogenic differentiation on day 14. The knockdown of *IGF2BP3* was confirmed by **(A)** qRT-PCR and **(B)** Western blot analyses. Gene expression was normalized using *GAPDH*. Mean value *±* standard deviation of at least three independent experiments. Representative images are shown from transfection control (Control) and *IGF2BP3* knockdown (IGF2BP3-KD) from two independent experiments. Beta-actin was used as a loading control. Molecular weight is shown in Kilodaltons (KDa). **(C)** Relative expression levels of the osteogenic genes *ALPL*, *RUNX2*, and *BGLAP* were investigated by qRT-PCR during osteogenic differentiation on days 7 and 14. Gene expression was normalized using *GAPDH*. Expression levels were calculated relative to the control on day 7. Mean value *±* standard deviation of at least three independent experiments. **(D)** Changes in osteogenic differentiation were investigated by Alizarin Red S staining on day 14. Representative images are shown from transfection control (Control) and *IGF2BP3* knockdown (IGF2BP3-KD) cultured with osteogenic media. Images are at 10X magnification. **p ≦ 0.01; two-tailed Student’s t-test compared to each respective control

The knockdown of *IGF2BP3* was confirmed by qRT-PCR and Western blot compared to the scramble control 48 h after transfection (qRT-PCR: Control: 1.0 *±* 0.0; IGF2BP3-KD: 0.3 *±* 0.1; *p* = 0.002; Fig. 6A-B). In contrast to IGF2BP1-KD, IGF2BP3-KD moderately downregulated the expression of the *ALPL* gene on days 7 and 14 of osteogenic differentiation (Day 7: Control: 3.3 *±* 3.5; IGF2BP3-KD: 1.8 *±* 2.3; *p* = 0.58 and Day 14: Control: 30.1 *±* 23.5; IGF2BP3-KD: 12.7 *±* 9.0; *p* = 0.21; Fig. 6C), while *RUNX2* remained mostly unchanged (Day 7: Control: 1.0 *±* 0.1; IGF2BP3-KD: 1.0 *±* 0.4; *p* = 1.00 and Day 14: Control: 0.7 *±* 0.5; IGF2BP3-KD: 0.8 *±* 0.2; *p* = 0.79; Fig. 6C) and *BGLAP* was slightly upregulated on day 7 and unchanged on day 14 compared to the scramble control (Day 7: Control: 1.1 *±* 0.5; IGF2BP3-KD: 2.0 *±* 0.7; *p* = 0.42 and Day 14: Control: 0.8 *±* 0.5; IGF2BP3-KD: 0.7 *±* 0.2; *p* = 0.59; Fig. 6C). Finally, no differences were observed in the Alizarin Red S staining from IGF2BP3-KD compared to the control on osteogenic differentiation day 14 (Fig. 6D).

## Discussion

Small- and long-non-coding RNAs, as well as RNA-binding proteins, are among the most well-known post-transcriptional regulators of gene expression. MicroRNAs (miRNAs) are highly conserved across evolution and involved in the post-transcriptional regulation of their target messenger RNAs (mRNAs) [1] with cellular effects that are both temporal and cell-tissue specific [1].

Previous studies have shown the importance of miRNAs during the osteogenic differentiation of mesenchymal stem cells (MSCs) as well as the existence of an ‘osteomiR’ expression signature that may be essential for the osteogenic differentiation of human amniotic membrane-derived MSCs [22, 23]. The highly conserved *let-7* family of miRNAs consists of 12 genes that encode for 9 mature miRNAs (*let-7a*, *let-7b*, *let-7c*, *let-7d*, *let-7e*, *let-7f*, *let-7 g*, *let-7i*, and *miR-98*) in humans. The expression of mature *let-7* miRNAs can be regulated at the transcriptional and post-transcriptional levels, where post-transcriptional repression of *pri-let-7* or *pre-let-*7 is mainly mediated by the RNA-binding protein LIN28 [18]. In humans, multiple components of the *let-7* cascade are developmentally regulated during the fetal-to-adult transition and following a post-traumatic event [5, 24, 25]. We have previously shown that some *let-7* miRNA family members are upregulated following traumatic extremity injury in patients who developed ectopic bone compared with patients who did not develop ectopic bone [5]. Moreover, *let-7c* and *let-7i* have been shown to positively regulate osteogenic differentiation and negatively regulate adipogenic differentiation of human adipose-derived MSCs by repressing the downstream target *HMGA2* [3], suggesting that *HMGA2* is a negative regulator of bone formation and a positive regulator of adipogenesis [3]. Finally, increased levels of *let-7c* induced osteogenic differentiation in rat dental pulp stem cells in vitro and in vivo by inhibiting *HMGA2* and the PI3K-Akt pathway [4].

In this study, we investigated the expression patterns of four *let-7* miRNA family members (*let-7a*, *let-7b*, *let-7d*, and *let-7f*) as well as the RNA-binding proteins *IGF2BP1-3*, which are known downstream targets of the *let-7* family of miRNAs [6, 8, 9]. The selection of the *let-7* miRNAs investigated in this study was based on the parameters detailed above (results section). Our results demonstrated that *let-7a* and *let-7b* had a similar expression pattern to each other (slightly upregulated on osteogenic differentiation day 7, downregulated on day 14, and slightly upregulated or unchanged on days 21 and 28), while *let-7d* and *let-7f* had similar expression patterns to each other and were consistently mildly upregulated in all timepoints (days 7, 14, 21 and 28) during human in vitro osteogenic differentiation. Expression of *let-7a* was shown to be downregulated in samples from patients with osteoporotic fracture, and upregulation of *let-7a* promoted bone morphogenic protein 2 (BMP-2)-induced osteoblastic differentiation of C2C12 cells [26]. Similar to our findings, *let-7a* was shown to be significantly downregulated at the halfway timepoint of FK506-induced osteogenic differentiation in rat bone marrow stromal cells compared to control cells [27]. Of note, osteogenic differentiation was induced for a total time of 14 days in the Zhang et al. study (as such, the halfway timepoint was day 7 [27]), while osteogenic differentiation was induced for a total of 28 days in this study (where the halfway timepoint was day 14).

Conversely, *let-7b* miRNA expression may be modulated depending on the cell type and/or differentiation protocol; *let-7b* was shown to be upregulated during estradiol-17β-induced osteogenic differentiation in rat primary BM-MSCs [19], while *let-7b* expression was downregulated during the osteogenic differentiation of human primary stem cells from the apical papilla [20]. Expression of both *let-7a* and *let-7b* was upregulated in pooled MSC cultures as well as significantly upregulated in MSCs from a biologically distinct donor at osteogenic differentiation day 7 compared to undifferentiated samples [28]. Of note, in the Goff et al. study [28], osteogenic differentiation was performed for up to 22 days, and while differentiation day 7 was considered an early timepoint, it is not necessarily comparable to day 7 in our study (where osteogenic differentiation was induced for up to 28 days). Moreover, unlike our study where the expression patterns between *let-7a* and *let-7b* where similar to each other but slightly distinct than *let-7d* and *let-7f*, Oskowitz et al. [29] demonstrated that *let-7a*, *let-7b*,* let-7d* and *let-7f* had all similar low expression levels at days 1 and 3 and high expression levels at days 7 and 14 of human multipotent stromal cells osteogenic differentiation compared to control samples [29]. Importantly, in the Oskowitz et al. study [29], days 1 and 3 were considered early osteogenic differentiation timepoints and days 7 and 14 were considered late osteogenic differentiation timepoints, suggesting differences in the osteogenic differentiation protocols between their study and this study.

Of interest, *let-7d* was shown to be expressed at low levels in the femur from mouse embryos (days 15 and 21 of gestation), while it was significantly upregulated in the femur of 3-week and 4-week-old male mice [3]. *Let-7d* was also significantly upregulated on day 6 of osteogenic differentiation in human adipose-derived MSCs [3]. Consistent with playing a role in osteogenic differentiation, Chang et al. [30]. reported that *let-7d* was upregulated during the first 3 days of osteogenic differentiation, as well as that overexpression of *let-7d* increased the activity of *ALPL* in immortalized human BM-MSCs.

Finally, *let-7f* was shown to be downregulated in the first 5 days of osteogenic differentiation of murine BM-MSCs in the presence of Dexamethasone [31]. In comparison, our study showed that both *let-7d* and *let-7f* were mildly upregulated on days 7, 14, 21, and 28 during human BM-MSC osteogenic differentiation. Altogether, our results demonstrate similar expression patterns for *let-7a* and *let-7b* as well as for *let-7d* and *let-7f* during human osteogenic differentiation, which may indicate unique functional roles during the processes of osteogenic commitment and differentiation of human BM-MSCs.

In humans, the insulin-like growth factor 2 mRNA-binding protein (IGF2BP) family comprises the *IGF2BP1*,* IGF2BP2*, and *IGF2BP3* genes. IGF2BP1-3 are RNA-binding proteins that have a wide range of cellular functions, including post-transcriptional regulation of their target transcripts through effects on RNA stability and translation [7]. *IGF2BP1-3* family members are known and experimentally validated targets of the *let-7* miRNAs [6, 8, 9]. In this study, we show that *IGF2BP1-3* genes have consistently low or downregulated expression levels in publicly available gene expression datasets from patients with different types of ectopic bone formation. In addition, during human in vitro osteogenic differentiation, our results demonstrate a similar expression pattern for *IGF2BP1* and *IGF2BP2* (mildly modulated or unchanged during osteogenic differentiation days 7, 21, and 28, and significantly downregulated on day 14), while *IGF2BP3* was mildly upregulated on day 7, and slightly downregulated on days 14, 21, and 28. Recently, the expression of *IGF2BP2* mRNA and protein was shown to be significantly upregulated on osteogenic differentiation day 7, unchanged on day 14, and significantly downregulated on day 21 in MC3T3-E1 subclone 14 cells compared to day 0 (osteogenic differentiation was reported for up to 21 days) [32]. Of interest, although there are *let-7* miRNA family members constantly expressed during human in vitro osteogenic differentiation, the expression of the *IGF2BP1-3* genes, which are validated targets of the *let-7* miRNAs, is not continuously repressed (compare Figs. 2 and 3).

Based on these results and to investigate the potential functional role of the *IGF2BP1-3* genes during human osteogenic differentiation, we performed knockdown studies in BM-MSCs immediately before osteogenic induction to investigate if the knockdown of any of the *IGF2BP1-3* genes had the potential to enhance osteogenic differentiation. We chose to directly target *IGF2BP1-3* transcripts to minimize the effects of any other variables (for example, the effects of other *let-7* targets – in addition to *IGF2BP1-3* – playing a role during osteogenic differentiation if direct *let-7* knockdown or *let-7* miRNA mimics approaches were used), and we chose to investigate the effects of the *IGF2BP1-3* knockdowns after days 7 and 14 of osteogenic differentiation (i.e., up to the halfway point of our 28-day osteogenic differentiation protocol [5, 11–13] to assess if changes in the *IGF2BP1-3* expression levels immediately before the start of osteogenic differentiation would affect BM-MSCs’ early commitment and/or differentiation choices. Our results demonstrated that the knockdown of *IGF2BP1* increased the levels of *ALPL* on days 7 and 14, while *RUNX2* and *BGLAP* were mildly increased on day 7 and decreased on day 14. Moreover, knockdown of *IGF2BP1* increased the level of calcium deposit on day 14 of osteogenic differentiation, while knockdown of the *IGF2BP2-3* genes had no major effects on osteogenic differentiation.

IGF2BP1 has been shown to play a role in osteogenic differentiation as a reader of the RNA modification N6-methyladenosine (m6A) methylation modification; m6A methylation is an extensively distributed modification found in eukaryotic RNA, and IGF2BP1, but interestingly not IGF2BP2, was shown to modulate the m6A methyltransferase METTL14-SMAD1 axis [33]. METTL14 has also been shown to promote in vitro osteogenic differentiation and in vivo bone formation in mice through the increased mRNA stabilization, mediated by the IGF2BP1-3 proteins, of the METTL14 downstream target *Beclin-1* [34]. The m6A methyltransferase METTL13 was also shown to promote osteogenic differentiation in human periodontal ligament cells and BM-MSCs through the increased stability of METTL13 downstream mRNA targets, which was shown to be mediated by the IGF2BP1 protein [34, 35]. Our observation that *IGF2BP1* may enhance the osteogenic commitment can reflect a stage- and/or lineage-specific function. Temporal downregulation of *IGF2BP1* may represent an active component of the human osteogenic program. Our results support a model where *IGF2BP1* repression might play a role in enhanced osteogenic lineage commitment.

The IGF2BP2 protein was shown to promote the osteogenic differentiation of BM-MSCs by binding to the circular RNA *circ-Plod2* and destabilizing *Mpo* transcripts, which was shown to be essential for the *circ-Plod2*-induced osteogenic differentiation [36]. Of particular interest to our study, knockdown of *IGF2BP2* in MC3T3-E1 cells demonstrated a significant increase in the activity of *ALPL* compared to the transfection control at osteogenic differentiation day 7, as well as a modest – but significant – increase in the levels of calcium deposits by Alizarin Red S staining compared to the control at osteogenic differentiation day 14 [32]. Of importance for comparison, osteogenic differentiation in the Zhou et al. study [32] was reported for up to 21 days in mouse MC3T3-E1 cells. Mechanistically, Zhou et al. [32]. identified that the IGF2BP2 protein promoted osteogenic differentiation by stabilizing *SRF* transcripts. Moreover, the RNA-binding protein *LIN28* was shown to inhibit the osteogenic differentiation of primary human dental pulp stem cells (hDPSCs), while its downstream target *let-7b* was shown to positively regulate the osteogenic differentiation of hDPSCs [10]. Mechanistically, it was found that *let-7b* directly binds and regulates *IGF2BP2* in hDPSCs, and knockdown of *IGF2BP2* promoted osteogenic differentiation as evidenced by increased levels of calcium deposits by Alizarin Red S staining compared to the transfection control in hDPSCs [10]. Osteogenic differentiation in the Yan et al. study [10] was reported for up to 21 days in hDPSCs. In our study, following the knockdown of *IGF2BP2* in human BM-MSCs, we observed only a mild modulation of *ALPL*, *RUNX2*, and *BGLAP* on days 7 and 14 of osteogenic differentiation; however, we did not observe effects on the levels of calcium deposits on osteogenic differentiation day 14. This difference might be a result of the distinct cell types, osteogenic differentiation protocols, and/or timepoints used in each study.

Furthermore, lentiviral-mediated inhibition of IGF2BP3 in human BM-MSCs decreased Alizarin Red S staining and, similar to our study, the levels of *ALPL* compared to the control in cells cultured in osteogenic media for 14 days [37]. Mechanistically, the IGF2BP3 protein was shown to promote osteogenic differentiation in human BM-MSCs by forming an RNA-protein complex with circular RNA *circ_AFF4* and stabilizing *FNDC5* mRNA [37]. More recently, circular RNA *circEIF4B* was shown to be significantly upregulated in human primary BM-MSCs at phytic acid-induced osteogenic differentiation in a high-glucose cell culture environment on days 7 and 14 (compared to high-glucose conditions without phytic acid) and formed a *circEIF4B*-IGF2BP3 complex to stabilize *ITGA5* mRNA and promote osteogenic differentiation [38].

Interestingly, Park et al. [39]. reported that intracellular delivery of Lin28A, a RNA-binding protein known to inhibit the processing of *let-7* miRNAs, fused with 30Kc19α as a cell-penetrating and protein-stabilizing protein, enhanced human urine-derived stem cells’ osteoblastic differentiation. Moreover, Lee et al. [40]. reported that the use of antisense oligonucleotides to inhibit *let-7* miRNAs, mimicking the physiological effects of LIN28 activation, enhanced the osteogenic differentiation potential of mesenchymal stromal cells. As such, despite our increased understanding of the role of non-coding RNAs in osteogenic differentiation and the elucidation of a miRNA signature that regulates osteogenic differentiation as well as post-traumatic ectopic bone formation [5, 21, 41], a better understanding of the underlying molecular mechanisms and pathways necessary for creating a tissue-specific phenotype remains the subject of multiple lines of investigation and recent research studies.

A constraint of our study that limits the generalizability of our results is that we used one lot of commercially available immortalized human BM-MSCs. As such, future studies will be required to validate these findings across additional human BM-MSC cells. Future studies could also aim directly at *let-7* knockdown experiments, followed by osteogenic differentiation to experimentally validate our findings. However, it is well-known that the *let-7* miRNAs have multiple downstream targets, which may add significant biological complexity to the experimental design and interpretation of the results. As such, *let-7* knockdown experiments combined with individual *IGF2BP1*, *IGF2BP2*, or *IGF2BP3* depletion would be more informative to resolve isoform-specific roles during human osteogenic differentiation.

In summary, we characterized the differential expression of specific members of the *let-7-IGF2BP1-3* regulatory axis during human in vitro osteogenic differentiation. Our results demonstrated that the *IGF2BP1* gene may enhance the osteogenic commitment of human BM-MSCs induced to in vitro osteogenic differentiation. Altogether, our results address aspects of unique importance to human in vitro osteogenic differentiation, including novel mechanistic insights in the *IGF2BP1*-mediated biology of human osteogenic commitment.

## Supplementary Information

Below is the link to the electronic supplementary material.

Supplementary Material 1

## Data Availability

The datasets generated during this study are included in this article and its supplementary files. The publicly available gene expression datasets (series GSE48129 and GSE94683) from patients with different types of ectopic bone formation analyzed in this study are available at the Gene Expression Omnibus data repository at https://www.ncbi.nlm.nih.gov/geo/query/acc.cgi?acc=GSE48129 and https://www.ncbi.nlm.nih.gov/geo/query/acc.cgi?acc=GSE94683 respectively.

## Abbreviations

Akt: Protein kinase B
BM-MSCs: Bone-marrow mesenchymal stem cells
cDNA: Complementary deoxyribonucleic acid
DMEM: Dulbecco’s modified eagle medium
FBS: Fetal bovine serum
HMGA2: High-mobility group AT-hook 2 (gene)
hDPSCs: Human dental pulp stem cells
IBC: Institutional Biosafety Committee
IGF2BP: Insulin-like growth factor 2 binding protein
IGF2BP1: Insulin-like growth factor 2 binding protein 1
IGF2BP2: Insulin-like growth factor 2 binding protein 2
IGF2BP3: Insulin-like growth factor 2 binding protein 3
KD: Knockdown
MSCs: Mesenchymal stem cells
mRNA: Messenger ribonucleic acid
miRNAs: Micro ribonucleic acids
m6A: N6-methyladenosine
NCBI: National Center for Biotechnology Information
PS: Penicillin/Streptomycin
PI3K: Phosphoinositide 3-kinase
qRT-PCR: Quantitative reverse-transcription polymerase chain reaction
siRNAs: Small interfering ribonucleic acids
