# Supplementary material for: Expression Profile and Role of the IGF2BP1-3 Genes During Human in vitro Osteogenic Differentiation
Source: Cell Biochem Biophys. 2026 Mar 9;84(2):2747–61. doi: 10.1007/s12013-026-02033-z (PMC13233644; doi:10.1007/s12013-026-02033-z)
Supplement: Supplementary file 1 — Supplementary Material 1 [file 12013_2026_2033_MOESM1_ESM.docx]

**Additional File 1 –** Differentially expressed levels (log fold change) of the *IGF2BP1-3* genes in patients with ectopic bone formation.

| **Study 1:** Characterization of osteogenic differentiation and gene expression profile of multiple miliary osteoma cutis.  **Study 1 information:** Series **GSE48129** available at the Gene Expression Omnibus data repository, performed on skin biopsies taken from patients with multiple miliary osteoma cutis, which is characterized by the formation of ectopic bone in the dermis and subcutis.  **Organism:** *Homo sapiens.*  **Expression profiling type:** Array.  **Samples analyzed:** GSM1169169, GSM1169170, GSM1169172, GSM1169166 and GSM1169174. | | |
| --- | --- | --- |
| **ID** | **Gene Symbol** | **Log Fold Change** |
| 223689_at | *IGF2BP1* | -2.336 |
| 241574_s_at | *IGF2BP1* | -1.082 |
| 227377_at | *IGF2BP1* | -0.263 |
| 218847_at | *IGF2BP2* | -0.474 |
| 223963_s_at | *IGF2BP2* | -0.970 |
| 1569033_at | *IGF2BP3* | -1.073 |
| 216493_s_at | *IGF2BP3* | -3.165 |
| 203819_s_at | *IGF2BP3* | -0.563 |
| 203820_s_at | *IGF2BP3* | -0.040 |
| **Study 2:** Transcriptome analysis of heterotopic ossification mesenchymal stromal cells.  **Study 2 information:** Series **GSE94683** available at the Gene Expression Omnibus data repository, performed on mesenchymal stromal cells from heterotopic ossification patients expanded *in vitro* for 2 passages.  **Organism:** *Homo sapiens.*  **Expression profiling type:** Array.  **Samples analyzed:** GSM2480834, GSM2480835, GSM2480836, GSM2480837, GSM2480838, GSM2480839, GSM2480840, GSM2480841, GSM2480842, GSM2480843, GSM2480844, GSM2480845, GSM2480846, GSM2480847, GSM2480848 and GSM2480849. | | |
| **ID** | **Gene Symbol** | **Log Fold Change** |
| 10923, 14874, 21176, 23872, 25441, 26461, 29923, 30562, 39082, and 43467 | *IGF2BP1* | -0.747 |
| 17552 | *IGF2BP1* | -0.532 |
| 22804 | *IGF2BP2* | 0.135 |
| 16034 | *IGF2BP2* | -0.196 |
| 35731 | *IGF2BP3* | -2.452 |
